# Analysis of the Effect of Concentrations of Four Whitening Products in Cover Transmissivity of Mediterranean Greenhouses

**DOI:** 10.3390/ijerph16060958

**Published:** 2019-03-18

**Authors:** Alejandro López-Martínez, Diego Luis Valera-Martínez, Francisco Domingo Molina-Aiz, María de los Ángeles Moreno-Teruel, Araceli Peña-Fernández, Karlos Emmanuel Espinoza-Ramos

**Affiliations:** 1 CIAIMBITAL Research Centre, University of Almería, Ctra. de Sacramento s/n, 04120 Almería, Spain; alexlopez@ual.es (A.L.-M.); fmolina@ual.es (F.D.M.-A.); mtm789@ual.es (M.d.l.Á.M.-T.); apfernan@ual.es (A.P.-F.); 2 Departmento de Ingenierías, Centro Universitario de la Costa Sur, Universidad de Guadalajara. Av. Independencia Nacional 151, Autlán de Navarro, Jalisco 48900, Mexico; karlos.espinoza@academicos.udg.mx

**Keywords:** greenhouse, agricultural solar protector, crop protection, cover transmissivity

## Abstract

The present work analyses the traditional method of applying whitening products on Mediterranean greenhouses. Four commercial whitening products (agricultural solar protectors, ASPs), applied at four doses, were compared with a non-whitened cover. The traditional product “Blanco de España” with 99% calcium carbonate (CaCO_3_) and other three products with 97% CaCO_3_ that incorporate adhesives were tested. The use of adhesives in ASP did not influence the effect of the different products on the inside temperature, and at the same dose all four products show a similar behaviour. The findings support the maximum dose recommended by other authors of 0.50 kg L^−1^ (50/100), above which the transmissivity of the greenhouse cover decreases by over 50%. The effect of ASP on the transmissivity of the cover depends principally on the dose applied, but also on the climatic conditions (solar radiation, cloud cover, etc.) and on the time of year (solar elevation). The habitual use of a constant dose throughout the year does not seem to be the most adequate. Recommended doses should vary according to the time of year and the desired degree of transmissivity reduction. The adhesive components are shown to provide a high degree of protection against heavy rain. The study recommends a standardised method of *ASP* application, establishing a method that allows the grower to verify the concentration of the product that will remain on the greenhouse cover.

## 1. Introduction

The success of the greenhouses in the province of Almería (Spain) is founded on low-cost structures and a temperate climate that permit relatively high yields. However, at certain times of the year natural ventilation does not suffice to combat the high temperatures, and consequently 99% of growers whiten the greenhouse cover [1]. To do so they apply a mixture of water and micronized calcium carbonate (“Blanco de España”). Despite the importance of this technique in the climate control of Mediterranean greenhouses, few technical or scientific works have studied this topic. Transmissivity of greenhouse cover is one of the main parameters influencing the energy balance that determine inside temperature, that can vary along a crop season between 0.44 and 0.80, depending on whitening [2]. Transmissivity of greenhouse cover with whitening is difficult to determine because it depends on the dose [3].

In hot and warm climates, shading is necessary in summer to reduce the solar radiation load in the greenhouses. Excess solar radiation can produce undesirable increases of temperature inside the greenhouse negatively affecting plants’ growth and direct damage on fruits (sunburn). A theoretical investigation carried out by writing energy and mass balance equations revealed that a whitened greenhouse cover significantly reduced both inside air and plant canopy temperatures [4]. A trial performed in Southern Spain with a pepper crop demonstrated that the use of whitening increased the commercial yield and reduced the incidence of sunburn [5]. Internal shading generates however a considerable amount of thermal radiation heat load that needs to be removed via cooling systems [6]. An important advantage of whitening with respect to the use of the internal shading screens is that it does not affect the ventilation of the greenhouse [7]. Gázquez et al. [8] observed that with a fully developed crop the combination of whitening and natural ventilation was the most efficient cooling strategy. They highlighted the problem need of determining the efficiency of the different whitening products and the optimum dose [8].

Kittas et al. [9] analysed different shading systems. Whitening slightly improved the proportion of photosynthetically active radiation (PAR) inside the greenhouse, reducing the proportion of infrared radiation. However, this technique has the drawback of providing less PAR uniformity than shading mesh, and its performance depends on outside climatic conditions of rain, humidity, etc. [10]. Baille et al. [11] studied the microclimate in a Greek glasshouse with a roof vent without whitening and with whitening. The transmissivity of the greenhouse cover decreased from 0.62 without whitening to 0.31 with whitening, and a similar percentage of decrease was obtained by Abreu and Meneses [12]. Baille et al. [11] also found a decreased stress level of a rose crop after whitening and an 18% increase in crop transpiration. The authors deemed the applied dose suitable, as a reduction in transmissivity of the greenhouse cover of over 50% would be excessive.

A beneficial effect of whitening is that it increases diffuse radiation inside the greenhouse [13]. In Shanghai (China), Luo et al. [14] applied a predictive model and found that crop biomass production was maximal when whitening reduced the greenhouse cover transmissivity by 10%. In Zimbabwe, Mashonjowa et al. [15] analysed, using a climatic model, the effect of whitening and of the accumulation of dirt on the transmissivity of a greenhouse cover. They observed that this technique significantly reduces maximum inside temperature, the vapour pressure deficit, the temperature difference between the crop and the surrounding air, and the crop transpiration rate, all of which help to avoid situations of crop stress.

A wide variety of whitening products are currently marketed under different commercial names. The principal component of all of them is calcium carbonate (CaCO_3_). Some commercial products can incorporate additives to improve its adherence to the greenhouse cover and to increase its resistance to weather conditions such as rain, while other additives can modify its optical characteristics. The main aim of the present work is to evaluate the traditional method of applying whitening products on the cover of a Mediterranean greenhouse, in comparison with different doses of application of four commercial whitening products (agricultural solar protectors, ASPs): the traditional product “Blanco de España”, *ASP_BE_*, and three other products which incorporate adhesives that provide greater resistance to rain. The experiments analysed the effect of these products on the transmissivity of the cover and on the temperature inside the greenhouse.

## 2. Materials and Methods 

### 2.1. Characteristics of the Experimental Greenhouse

**Figure 1:**
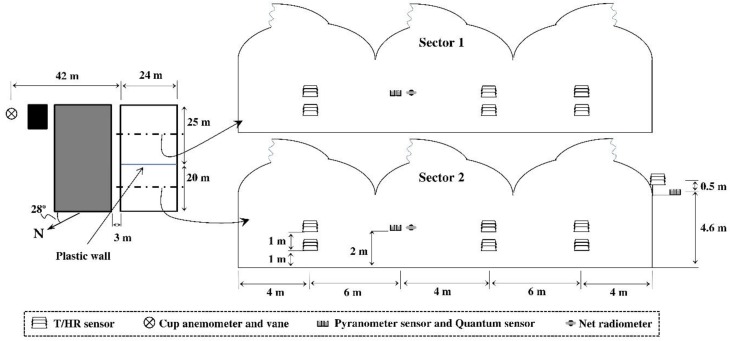
Position of the greenhouse at the experimental farm and location of the sensors. (Adapted of López et al. [16]).

The experiments were carried out in an empty multi-span Mediterranean greenhouse (24 × 45 m^2^) with three roof vents, located at the “Catedrático Eduardo Fernández” farm of the UAL-ANECOOP Foundation (36° 51′ N, 2° 16′ W and 87 MASL) in the province of Almería in Southern Spain. The greenhouse is permanently divided into two sectors by an interior plastic wall (Figure 1); sectors 1 (East) and 2 (West) measuring 24 × 25 m^2^ and 24 × 20 m^2^, respectively. The side walls of the greenhouse consist of undulating strips of rigid polycarbonate, while the roof of the greenhouse was covered with TRIPLAST three-layer co-extrusion greenhouse film (PE-EVA-PE) of 0.2 mm thickness (Plastimer-Morero & Vallejo Industrial, Almería, Spain). The manufacturer describes the technical characteristics of the cover as diffuse colourless, 200 µm thickness, 85% transmissivity to visible light, 50% transmissivity to diffuse light and 8% transmittance to infrared light.

The greenhouse is fitted with three roof vents measuring 40 × 1 m^2^ each (22.5 × 1 m^2^ in sector 1 and 17.5 × 1 m^2^ in sector 2), with the same orientation to the wind in each sector. The ventilation surface, i.e. surface area of the vent openings/greenhouse area, or *S_V_*/*S_A_*, was 11.25% for sector 1 and 10.81% for sector 2. The roof vents were fitted with insect-proof screens with a thread density of 10 × 20 threads cm^−2^ (36.0% porosity) and with the following geometric characteristics: thread density measured 9.6 × 20.3 threads cm^−2^; weft pore length 239.9 ± 18.5 µm; warp pore length 765.4 ± 27.1 µm; thread diameter 259.6 ± 19.1 µm; diameter of the inside pore circumference 241.9 ± 19.1 µm; mean pore area 0.182 ± 0.015 mm^2^; corresponding with screen 3 discussed in López et al. [16]. 

### 2.2. Measurement Equipment inside the Greenhouse

Temperature and relative air humidity were measured inside and outside the greenhouse by means of 13 CS215 sensors (Campbell Scientific Spain S.L., Barcelona, Spain) with accuracy for temperature of ±0.4 °C over 5–40 °C and for relative humidity of ±2% over 10%–90% RH. The sensors were protected from radiation inside a naturally aspirated box 41003-5 (Campbell Scientific Spain S.L., Barcelona, Spain). The data of humidity did not differ between the two sectors of the greenhouse, as experiments were conducted without crop.

Solar radiation and PAR were measured inside and outside the greenhouse with three SP1110 pyranometers (Campbell Scientific Spain S.L.; sensitivity range of 350–1100 nm; accuracy of ±5%; Barcelona, Spain) and with three quantum sensors SKP215 (Skye Instruments Ltd, Llandrindod Wells, UK; sensitive to light between 400 nm and 700 nm wavelength; measurement range of 0–5 × 10^4^ µmol m^−2^ s^−1^; accuracy ±5%). Net radiation was measured inside the greenhouse with two NR-Lite2 net radiometers (Kipp & Zonen B.V., Delft, The Netherlands; spectral response: 0 to 100 μm; measurement range of ±2000 W m^−2^; accuracy of ±5%). The data from all sensors were stored in five CR3000 microloggers (Campbell Scientific Spain S.L.) with a frequency of 1 Hz. Outside wind speed was measured at 10 m height with a Meteostation II (Hortimax S.L., Almería, Spain) incorporating a cup anemometer (measurement range of 0 to 40 m s^−1^; accuracy of ±5%) and a vane for wind direction (accuracy ±5°). The Meteostation II measurements were stored in an independent computer system once a minute. Figure 1 presents the location of the sensors in the experimental greenhouse.

### 2.3. Experimental Design

Data were taken in the months of July, August, September and October 2014 (Table 1). Sector 1 of the greenhouse without ASP was used as control. In sector 2, four *ASP* of different characteristics were applied: the traditional product “Blanco de España“ (*ASP_BE_*), and three products that incorporate adhesives, Flex (*ASP_F_*), SuperFlex (*ASP_SF_*) and Special Pepper (*ASP_SP_*), all of which are commercial products (Indaloblanc S.L., Almería, Spain). Three concentrations of each product were tested [kg of product / l of water]: 0.125 kg L^−1^ (25/200), 0.25 kg L^−1^ (25/100), 0.50 kg L^−1^ (50/100); a fourth concentration of 0.08 kg L^−1^ (25/300) was tested for the product *ASP_BE_*. The manufacturers recommend a dose of 0.25 kg L^−1^ (25/100), though a work published after the experiments were carried out found that the mean dose applied in the province of Almería is 40/100 [1], an intermediate value between 25/100 and 50/100 tested here.

Application of the product involves consuming approximately 0.1 l of mixture per m^2^ of the greenhouse cover, which implies the following approximate quantities of product: 8.3, 12.5, 25.0 and 50.0 g m^−2^ (for the four concentrations tested). The traditional method of applying these products follows three steps: (i) the product is mixed according to the dose (kg/l) in a container of large capacity; (ii) one worker operates the hydraulic pump to apply the mixture through a hose; (iii) a second worker holding the hose (without a regulated nipple) walks over the greenhouse roof, applying the quantity of product that he considers suitable. There is no technical control of the real quantity of product applied to the greenhouse cover. Rather it all depends on the skill and knowhow of the worker. 

According to the technical data supplied by the products’ distributors, the traditional product “Blanco de España” *ASP_BE_* consists of over 99% calcium carbonate (CaCO_3_), whereas the other three products (*ASP_F_*, *ASP_SF_* and *ASP_SP_*) have about 97% calcium carbonate (CaCO_3_). The traditional product *ASP_BE_* incorporates less than 1% of other elements, without adhesive substances. The others three products use unidentified adhesives and elements in proportions less than 3%. The manufacturer did not supply data on the precise compositions of additives, but indicate that *ASP_SF_* presents a higher resistance to weather elements like rain with an approximate durability of 3 to 5 months, whereas for *ASP_F_* and *ASP_SP_* it is about 3 months. 

**Figure 2:**
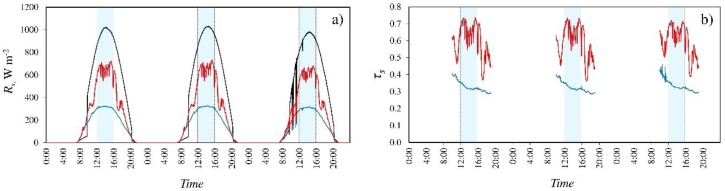
Levels of solar radiation (**a**) and values of transmissivity of the cover to solar radiation (**b**) on 02–04/08/2014. ―, exterior; ―, sector 1 (without *ASP_BE_*); ―, sector 2 (with *ASP_BE_* at a concentration of 0.50 kg L^−1^). Interval of 4 hours around the time when the sun is shining vertically (■).

All the products are soluble in cold water, with a mean and maximum particle diameters of 2.8 µm and 33 µm, respectively. Each dose of each product was tested over 5 days: the product was applied early in the morning on the first day and data were taken on the second, third and fourth days (used as the three repetitions for statistical analyses); on the fifth day, the cover was cleaned and the following dose was applied, commencing a new test cycle. The different concentrations of each product were tested in three consecutive days, to allow a minimum of 720 data when analysed statistically values of transmissivity in the interval 12–16 h. These three days can be considered as different replications (Figure 2) of each treatment (a product with a concentration). The climatic conditions outside the greenhouse on the days when data were recorded are presented in Table 1. For the first tests carried out with the product *ASP_BE_* (from July 19 to August 4, 2014) no inside temperature data are available due to a malfunction of the sensors, and so a second set of tests was carried out with this product in late September and early October. Tests were carried out at a time of year when crops are usually transplanted, i.e. when the cooling effect of crop evapotranspiration is low. As no crop was present in the greenhouse, the effect of the products was quantified in the most extreme conditions possible, simulating the situation when a crop is transplanted.

In spring 2015, an experiment was carried out to determine the resistance to rain of the traditional adhesive-free product *ASP_BE_* and of *ASP_F_*, which includes adhesive. On March 27 2015, these products were applied in sectors 1 and 2, respectively, of the experimental greenhouse at a concentration of 0.25 kg L^−1^ (25/100). From April to July the transmissivity of the greenhouse cover was determined in both sectors at different times in order to evaluate the effect of precipitation and time on the two products. Transmissivity was determined by measuring PAR outside the greenhouse and inside each sector. An HD2302.0 photo-radiometer (Delta OHM S.R.L., Padua, Italia) was used, equipped with an LP 471 PAR probe (sensitive to light between 400 nm and 700 nm wavelength; measurement range of 0.01 to 104 µmol m^−2^ s^−1^; accuracy <5%), to measure the photon flow in the PAR range.

### 2.4. Statistical Analysis

We have carried out regression analyses to compare the different variables for statistically significant relationships (*p*-value<0.05) using Statgraphics® Centurion 18 v 18.1 (Statgraphics Technologies, Inc., The Plains, VA, USA). The different transmissivity and inside air temperature in both compartments of the experimental greenhouse (with and without whitening) were examined using an analysis of variance (*p*-value < 0.05), comparing mean values using Fisher’s least significant difference (LSD) approach. When there was a difference statistically significant between the standard deviations, the parametric analysis was not viable by means of an analysis of variance. For parameters with different variance, we made a non-parametric analysis with the Friedman test, appropriate when each row represents a block (the date of measurement), using box-and-whisker plot [17].

## 3. Results and Discussion

The aim of this work was to know about the effect on transmissivity of using products composed mainly of CaCO_3_ at different concentration for whitening Mediterranean greenhouse roofs. The results obtained were statistically analyzed to verify the influence of different products on reduction of cover transmissivity and to compare different dose of each product. For a better understanding the results were divided and presented under four subsections. In Section 3.1 (Transmissivity of the cover with Agricultural Solar Protector without adhesives) we analyse differences in behavior of the use of traditional product for whitening Mediterranean greenhouse roofs *ASP_BE_* on two test periods, July-August and September-October. Transmissivity data for *ASP_BE_* are compared to the other products, *ASP_F_*, *ASP_SF_* and *ASP_SP_* incorporating adhesives in Section 3.2 (Transmissivity of the cover with Agricultural Solar Protector with adhesives). Section 3.3. (Effect of climatic conditions on the transmissivity of the cover with Agricultural Solar Protectors) show the effect of rain on the transmissivity of the whitened greenhouse cover, comparing the products *ASP_BE_* (without adhesive) and *ASP_F_* (with adhesive). Finally, a global analysis of the four products on the temperature inside the greenhouse is presented in Section 3.4 (Greenhouse temperature influenced by the cover with Agricultural Solar Protector).

### 3.1. Transmissivity of the Cover with Traditional Agricultural Solar Protector without Adhesives

Figure 2a shows the level of solar radiation outside and inside the greenhouse for the experiment carried out with *ASP_BE_* at a concentration of 0.50 kg L^−1^ on August 2–4, 2014. Irrespective of the dose applied, the use of this product has been seen to reduce fluctuations in the intensity of solar (Figure 2a) and PAR radiation inside the greenhouse, confirming the findings of Baille et al. [11]. This is beneficial for the crop, since the radiation levels received will remain stable throughout the day. The results of Baille et al. [11] showed that application of the product on the greenhouse cover reduced both the difference in temperature between crop leaves and the surrounding air and “the canopy-to-air vapour pressure deficit”, while increasing the crop transpiration rate, which mitigated the previously observed fluctuations in this parameter the day after application.

The values of transmissivity of the greenhouse cover fluctuate less when *ASP_BE_* is applied (Figure 2b). The combined effects of reduction of fluctuation of the mean inside radiation and of the calculated transmissivity are likely due to the increase in the proportion of diffuse radiation when *ASP* is used [13], as diffuse radiation is less sensitive to the presence of obstacles including the greenhouse structure itself and any greenhouse equipment [11]. Indeed, Baille et al. [11] found less fluctuation in the values of mean inside radiation and of transmissivity of the cover with *ASP* than without it (mean values of 0.31 and 0.62, respectively, from 9:00 to 19:00). Figure 2b illustrates the sharp fall in the transmissivity of the greenhouse cover in sector 1 in periods when the withdrawn shading mesh affected the radiation sensors (between 11:00 and 11:30, and 17:00 and 17:30, approximately).

**Figure 3:**
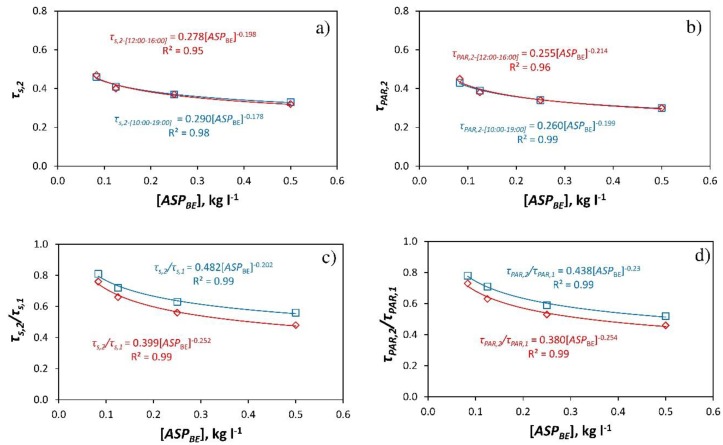
Mean values of transmissivity of the greenhouse cover for different concentrations of *ASP_BE_*. [*ASP_BE_*]; *τ_s_*, transmissivity to solar radiation (**a**); *τ_PAR_*, transmissivity to PAR (**b**). Mean values of the ratio *τ_s,2_*/*τ_s,1_* (**c**) y *τ_PAR,2_*/*τ_PAR,1_* (**d**). Subscript: *1*, sector 1 (without *ASP_BE_*); *2*, sector 2 (with *ASP_BE_*). □, 10:00 to 19:00; ◊, 12:00 to 16:00.

Table 2 presents the values of transmissivity of the greenhouse cover to solar radiation, *τ_s_* (*R_s,i_*/*R_s,o_*), and PAR, *τ_PAR_* (*R_PAR,i_*/*R_PAR,o_*), for each dose of product applied. Transmissivity was analysded between 12:00 and 16:00 h, obtaining the average value at the interval of 4 hours around the time when the sun is shining vertically (local time 14:30 h). For the climatic conditions of the experiments, the transmissivity of the cover to total radiation and PAR can be obtained from a power regression equation based on the dose applied (Figure 3a,b).

The power regression equations presented in Figure 3 are only valid for concentrations of *ASP_BE_* between 0.08 and 0.50 kg L^−1^; for concentrations close to 0 these fits are not valid, as the values obtained would tend to infinity. Figure 3c,d show the fits to obtain the ratio *τ_s,2_*/*τ_s,1_* as a function of the dose of *ASP_BE_* applied. The power regression equations presented in Figure 3c,d would be valid to estimate the effect on transmissivity of any type of greenhouse cover as a function of the concentration of *ASP_BE_* applied under similar climatic conditions to those of these experiments.

For all doses analysed, transmissivity of the whitened cover with *ASP_BE_* was statistically lower than transmissivity of the cover without whitening (Table 2). We can also observe a reduction statistically significant of transmissivity when the dose of whitening increased (Table 2). Furthermore, transmissivity of the un-whitened cover show a statistically significant variation along the year. At the end of July, transmissivity increased with the day of year (*DOY*), as we can observe in Table 2.

The dose of 0.50 kg L^−1^ (50/100) could be recommended as the maximum concentration, respecting the limit of 50% reduction in transmissivity recommended by Baille et al. [11]. In the present study, with this dose, the values of transmissivity of the cover were around 0.30, which is similar to the results obtained by the cited authors with a much lower concentration of the product, 0.08 kg L^−1^ (8/100). This discrepancy may be mainly due to: (i) the traditional method of applying the product, which is imprecise and unreliable, and as a result the amount of product that is finally applied to the cover will depend on the skill of the worker to a great extent; and (ii) the use of different types of greenhouse cover, namely a three-layer co-extrusion greenhouse film (PE-EVA-PE) of 0.2 mm in the present study and a glass roof in the case of Baille et al. [11].

Baille et al. [11] found that the ratio of net to solar irradiance measured above a well-developed crop of roses was not significantly different before and after whitening, with *R_n,i_*/*R_s,i_* values of 0.70 before application of the product and 0.73 afterwards. In the present study the greenhouse was empty, i.e. in similar conditions to a greenhouse with a recently transplanted crop, and in this case *R_n,i_*/*R_s,i_* was slightly lower with *ASP_BE_* (*R_n,1_*/*R_s,1_*) than without it (*R_n,2_*/*R_s,2_*), as Table 2 illustrates. *ASP_BE_* appears to reduce the amount of direct solar radiation entering the greenhouse, but it increases the proportion of diffuse radiation inside the greenhouse, which influences the lower receiver of the net radiation sensor. Were a crop present, maybe all this radiation would be recorded by the sensor, and no difference would be observed in the *R_n,i_*/*R_s,i_* ratios between sectors, as occurred in the above-mentioned study.

PAR is presented as µmolm^−2^s^−1^, and in order to compare it with the values of solar/total radiation obtained with a pyranometer (Wm^−2^) it can be multiplied by a factor of 4.57 (in µmolm^−2^ s^−1^)/(W m^−2^) [18] or 4.6 [19], the former of which was chosen. One drawback of using the traditional product *ASP_BE_* is that it slightly reduced the proportion of PAR vs. total radiation (*R_PAR_*/*R_s_*) inside the greenhouse (Table 2), which contrasts with the findings of Kittas et al. [9], who recorded a slight increase in this proportion. This type of product is 99% calcium carbonate (CaCO_3_), but other compounds should be sought to act selectively depending on the wavelength of the radiation.

In short, the use of *ASP_BE_*, applied in the traditional fashion, led to a marked reduction in the transmissivity of the greenhouse cover. On the downside, it also appeared to reduce slightly the proportion of net radiation (though it should be noted that there was no crop in the greenhouse) and the proportion of PAR with respect to mean total radiation. The reduction in transmissivity has been seen to be statistically related to the dose applied, although the values of transmissivity of the greenhouse cover below a certain dose of product also depend on the prevalent conditions of solar radiation and elevation (see Section 3.3). It should also be remarked that the doses recommended by manufacturers are difficult to adhere to, since the product application method precludes verification of the final number of grams of product per m^2^ of roof. 

Due to technical problems, no inside temperature data were available for the experiments carried out in summer with *ASP_BE_*, and so it was decided to repeat the experiments in early October omitting the lowest concentration of the product, 0.08 kg L^−1^. Soriano et al. [20] carried out a laboratory study on how the angle of incidence of solar radiation affected the transmissivity of several samples of glass, finding that the transmissivity was greatest when radiation was perpendicular to the glass. Transmissivity decreased with the angle of incidence, though the decrease was not marked until the angle reached 50–60° with respect to the perpendicular; Mashonjowa et al. [15] obtained similar results. Given these findings, it might be expected that the effect of *ASP_BE_* on transmissivity of the greenhouse cover would differ between the experiments carried out in summer and autumn. Furthermore, in Mediterranean greenhouses *ASP_BE_* is usually only applied on the roof, not on the sides, and so the effect of the product might be expected to increase with solar elevation. In addition to the effect of the angle of incidence of the radiation, the level of diffuse radiation will affect the transmissivity values calculated, leading to differences depending on whether the sky is clear or overcast.

Table 3 presents the transmissivity data obtained for the experiments carried out in September-October, and the values in sector 1 without *ASP* are higher than those in summer (Table 2). Moreover, the transmissivity of the cover for the same dose of product was significantly higher than that recorded in summer (Table 2 and Table 3). The transmissivity to solar radiation of the cover without *ASP_BE_* was 8% (10:00–19:00) and 10% (12:00–16:00) greater in autumn than in summer. When *ASP_BE_* was applied, between 12:00 and 16:00 transmissivity to solar radiation was 18% ([*ASP_BE_*] = 0.125 kg L^−1^), 19% ([*ASP_BE_*] = 0.25 kg L^−1^) and 20% ([*ASP_BE_*] = 0.125 kg L^−1^) greater in the autumn experiments (Table 2 and Table 3). As in the first experiment, transmissivity of whitened cover decreased (with statistical significance) when the dose of *ASP_BE_* increased. A statistical difference was also observed between whitened cover with *ASP_BE_* and un-whitened cover (Table 3). However, in autumn transmittance of the cover without whitening reduced along the date, inversely to that observed in summer (Table 2). This difference was not statistically significant for transmissivity around the time of maximum outside solar radiation (12:00–16:00).

This would appear to contradict the findings of other works [15,20], since solar elevation is greater in summer than in autumn, suggesting that transmissivity should also be greater. However, the mean angles of incidence of solar radiation on the greenhouse cover have been calculated (Section 3.3), and they are below 50–60°, the margin in which reduction in transmissivity becomes more marked. On the other hand, in autumn the degree of solar elevation is lower and so a greater proportion of total radiation in the greenhouse will enter through the sides, which will affect transmissivity values calculated. Finally, in autumn there is a greater probability of overcast skies, conditions in which the proportion of diffuse radiation is greater, which will contribute to higher transmissivity values calculated in autumn than in summer. 

This variation in transmissivity of the greenhouse cover, an in the effect of applying *ASP_BE_*, at different times of year (differences in solar elevation and the level of solar radiation) makes it difficult to compare the different *ASP* tested in the present work. It also makes it difficult for the manufacturers to suggest a recommended dose, since on the one hand the method of application would have to be standardised to ensure that the correct amount of product was applied to the greenhouse roof. On the other hand, the manufacturers’ recommendations should take into account different climatic conditions (time of year, level of radiation, etc.).

### 3.2. Transmissivity of the Cover with Agricultural Solar Protector with Adhesives

This product was tested in the first weeks of August, with high levels of solar radiation and outside temperature. The experiments using a concentration of 0.25 kg L^−1^ (25/100) took place on overcast days, which affected the results: the transmissivity values obtained were higher than those for the concentration of 0.125 kg L^−1^ (25/200) (Table 4). This may be due to the influence of the cloudy skies (a greater proportion of diffuse radiation) and to the traditional method of application, which makes it impossible to verify the exact quantity of product retained on the cover.

As for the standard product *ASP_BE_* (Table 2 and Table 3), the transmissivity of the cover with whitening product using adhesives in tis compositions (*ASP_F_*, *ASP_SF_* and *ASP_SP_*) was statically lower that the un-whitened cover, for all the doses tested (Table 4). In general, the increase in the dose produced a reduction (statically significant) of the transmissivity (Table 4). However, differences statistically significant between the two lower doses (0.125 and 0.25 kg L^−1^) changed in function of the date and the weather conditions (cloudy and sunny days).

The reduction of transmissivity with respect to the cover without whitening was statistically greater (lower values of the ratio *τ_s_,_2_*/*τ_s,1_*) whit the higher concentration of 0.50 kg L^−1^ (50/100) of the products *ASP_F_* and *ASP_SF_* than whit the others two doses or than whit the others products *ASP_BE_* and *ASP_SP_* (Table 5). 

With *ASP_F_* at the dose of 0.50 kg L^−1^, a far greater decrease in transmissivity was observed (*τ_s_,_2_*/*τ_s,1_* = 0.33 between 12:00 and 16:00) than with *ASP_BE_* (*τ_s,2_*/*τ_s,1_* = 0.48 in summer and 0.57 autumn) (Table 5). However, for the concentration of 0.125 kg L^−1^ the difference between *ASP_F_* (*τ_s,2_*/*τ_s,1_* = 0.73 between 12:00 and 16:00) and *ASP_BE_* (*τ_s,2_*/*τ_s,1_* = 0.66 in summer and 0.68 in autumn) was to the contrary, i.e. the decrease in transmissivity was greater with *ASP_BE_*. When comparing the results of these two products important factors should be taken into account: (i) the experiments were carried out on different days under similar but not identical climatic conditions; (ii) the traditional method of applying the products does not ensure that the same amount of product was applied per m^2^ of greenhouse cover in each replication or test, even though the dose kg L^−1^ was the same. The results do indicate, however, that the presence of adhesives in the product (less than 3%) clearly increases the effect of the product on the transmissivity of the cover.

The ratio *τ_s,2_*/*τ_s,1_* was also greater in autumn (Table 2 and Table 3): *τ_s,2_*/*τ_s,1_* was 3% ([*ASP_BE_*] = 0.125 kg L^−1^), 13% ([*ASP_BE_*] = 0.25 kg L^−1^) and 17% ([*ASP_BE_*] = 0.125 kg L^−1^) greater in the autumn experiments than in the summer ones (Table 5).

The difference between the products *ASP_F_* and *ASP_SF_* lies in the quantity of adhesive components they incorporate. Although the manufacturers declined to provide specific data, it is known that *ASP_SF_* has the greater adhesive content. These tests were carried out using concentrations of 0.125 kg L^−1^ and 0.50 kg L^−1^ on sunny days, and of 0.25 kg L^−1^ in partly cloudy conditions. For this product, the ratio *τ_s,2_*/*τ_s,1_* was similar at concentrations of 0.125 kg L^−1^ and 0.25 kg L^−1^ (Table 5), possibly due to the partially cloudy sky during the test for the latter concentration, which might explain the reduced effect of *ASP_SF_* on the transmissivity of the cover. In comparison with the results obtained for the traditional product *ASP_BE_*, there appear to be no statistical differences in the values of the ratio *τ_s,2_*/*τ_s,1_* (Table 2 and Table 4). Between 12:00 and 16:00 the ratio *τ_s,2_*/*τ_s,1_* reaches similar values at a concentration of of 0.25 kg L^−1^ for *ASP_SF_* (0.58) and for *ASP_BE_* (0.56) in summer). Only at the highest concentration tested for *ASP_SF_* (0.50 kg L^−1^) was a greater difference observed in the ratio *τ_s,2_*/*τ_s,1_* (0.48 for *ASP_BE_* in summer and 0.57 for *ASP_BE_* in autumn and 0.41 for *ASP_SF_*). As occurs with *ASP_F_*, with the product *ASP_SF_* (which in theory contains a greater quantity of adhesives) the results provide no clear indication that the adhesive clearly increases the effect of the product on the transmissivity of the greenhouse cover.

Of the products tested, *ASP_SP_* contains the largest amount of adhesives. For this product tests were carried out at concentrations of 0.125 kg L^−1^ and 0.25 kg L^−1^ on mainly sunny days, while at the concentration of 0.50 kg L^−1^ on the last two days of testing the sky was rather overcast. Comparison of the results obtained for *ASP_SP_*, tested in September, with those obtained in September/October for *ASP_BE_* does not highlight any great differences (Table 3 and Table 4). The lowest ratio *τ_s,2_*/*τ_s,1_* at concentrations of 0.125 kg L^−1^ was obtained for the *ASP_SP_*, and the highest for the *ASP_F_* with a statistical significant difference. For the higher dose of 0.50 kg L^−1^, we can observe the inverse effect, with the *ASP_SP_* producing the greatest value of the ratio *τ_s,2_*/*τ_s,1_*., and the *ASP_F_* the lowest (Table 5). This result confirm the difficulty to predict the behavior of the different whitening products. At greater doses, the product with most adhesive component can allow a better adherence to the plastic cover, requiring less quantity of product to cover the roof, resulting in a greater transmissivity that product with a lower adherence. However, at low doses the effect of the different type of adhesives could affect to the greenhouse transmissivity. 

### 3.3. Effect of Climatic Conditions on the Transmissivity of the Cover with Agricultural Solar Protectors 

**Figure 4:**
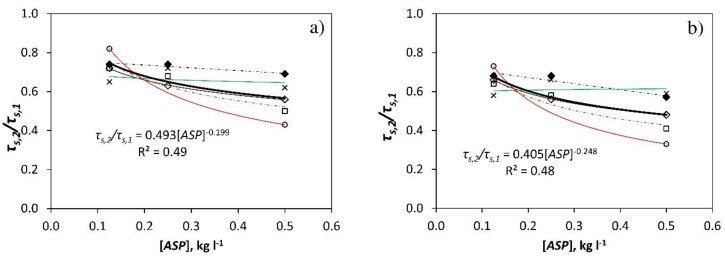
Mean values of the ratio *τ_s,2_*/*τ_s,1_* between 10:00 and 19:00 (**a**) and between 12:00 and 16:00 (**b**) according to the dose [kg L^−1^] of the four *ASP* tested: ◊ (―), *ASP_BE_* (summer); ♦ (-·-), *ASP_BE_* (autumn); ○ (―), *ASP_F_*; □ (-·-), *ASP_SF_*; × (―), *ASP_SP_*. *τ_s_*, transmissivity to solar radiation. Subscript: *1*, sector 1 (without *ASP*); *2*, sector 2 (with *ASP*). (**-**) setting curve considering all the products.

In short, Figure 4 illustrates that there were no notable differences between the capacity of the four products tested to reduce the transmissivity of the greenhouse cover (*τ_s,2_*/*τ_s,1_*) at low concentrations (0.125 kg L^−1^ and 0.25 kg L^−1^). Bearing in mind that CaCO_3_ constitutes 97-99% of the products, and that a maximum of 3% is composed of adhesives, we can state that the addition of this amount of adhesive does not noticeably alter the products’ effect on the transmissivity of the greenhouse cover for low doses. Considering that all 4 products behave in a similar fashion at the same concentration, we can obtain a setting curve with which to estimate the ratio *τ_s,2_*/*τ_s,1_* as a function of the dose applied [kg L^−1^].

Statistical analyses have been carried out considering all the products (*ASP_BE_*, *ASP_F_*, *ASP_SF_*, *ASP_SP_*) as the same *ASP*, in order to determine which of the parameters measured bear a significant influence on the values of transmissivity of the greenhouse cover with and without *ASP*.

The curved roof of the experimental greenhouse means that the angle of incidence of the radiation from the cover varies from practically 0° to 90° according to the position of the sun and the part of the roof considered. Considering a mean roof angle of 23.1° (calculated as the mean value of 50 different points in the roof), the angle of incidence of solar radiation ***α_c_*** for the southern slope of the cover at the time of maximum solar elevation would vary between 20.3° and 37.3° (for the experiments from July 19–21 and from October 10–12, 2014, respectively), and between 23.2° and 50.7° for the northern slope of the cover for the same experimental periods. For an angle of incidence where 0° corresponds to a perpendicular incidence of solar radiation and a value of 90° corresponds to incidence parallel to the cover. These mean angles of incidence do not reach 50–60°, beyond which Soriano et al. [20] found that transmissivity decreased significantly.

**Figure 5:**
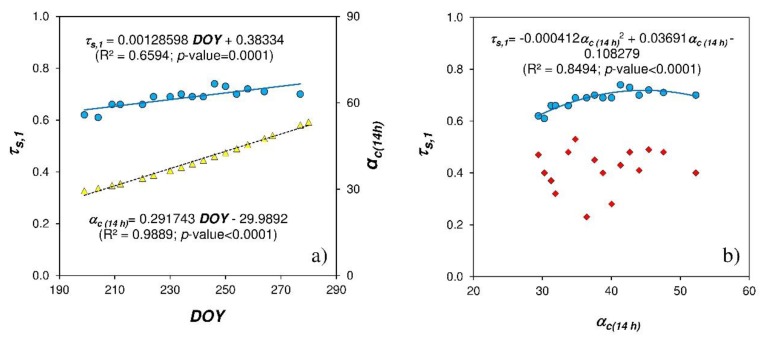
Evolution of the transmissivity *τ_s_*,*_1_* (•) of the cover greenhouse without whitening between 12:00 and 16:00 and of the angle of incidence of solar radiation *_c(14 h)_* (▲) for the northern slope at 14:00 h according to the day of the year *DOY* (**a**). Relationship between transmissivity of the cover without whitening *τ_s_*,*_1_* (•) and with the different *ASP* tested *τ_s_*,*_2_* (♦) in function of the angle of incidence *_c(14 h)_* of solar radiation. Regression curves for cover transmissivity (**▬**) and angle of incidence (---) (**b**).

The angle of incidence ***α_c (14 h)_*** obtained for the northern slope at 14 h, around the time of maximum solar elevation, increased along the period of tests avec the *DOY*, producing a variation of transmissivity *τ_s,1_* (Figure 5a). The influence of this angle in the cover transmissivity for the greenhouse without whitening (Figure 5b) can be represented by a statistically significate regression as (R^2^ = 0.85; *p*-value < 0.0001):

(1) $$\tau_{s,1}=-0.000412\propto_{c\left(14h\right)}^{2}+0.03691\propto_{c\left(14h\right)}-0.108279$$

Data analysis from all the tests carried out from July to October shows that there is a statistically significant correlation (*p*-value<0.01) between the transmissivity of the greenhouse cover in sector 1 (without *ASP*) (*τ_s,1_*), the maximum daytime solar elevation (*γ_max_*) and solar radiation. Analysis of the period from 10:00 to 19:00 provides the following equation (R^2^ = 0.54; *p*-value = 0.0068):

(2) $$\tau_{s,1}=0.83199-0.000526823\cdot \gamma_{max}-0.000369459\cdot R_{s,o}$$

Omitting solar elevation from (2), since the angles of incidence of solar radiation do not reach those beyond which Soriano et al. [20] found a sharp fall in transmissivity, provides the following equation with a lower *p*-value (R^2^ = 0.53; *p*-value = 0.0014):

(3) $$\tau_{s,1}=0.822674-0.000418176\cdot R_{s,o}$$

The transmissivity of the cover without *ASP* increases as solar radiation decreases, which may be due to the proportion of diffuse radiation on the days in which the level of radiation is lower (overcast days and/or autumn days). Between 12:00 and 16:00 the following equation is obtained (R^2^ = 0.36; *p*-value = 0.0137):

(4) $$\tau_{s,1}=0.830939-0.000172622\cdot R_{s,o}$$

Given the relationship between the transmissivity of the cover without *ASP* and the levels of outside radiation (cloud, diffuse radiation), it appears logical to suppose that the effect of applying any *ASP* product on the greenhouse cover will depend on, among other factors, solar radiation and the concentration or dose of the product [kg L^−1^]. Between 10:00 and 19:00 the following equation is obtained (R^2^ = 0.58; *p*-value = 0.0038):

(5) $$\tau_{s,2}/\tau_{s,1}\;=0.99093-0.000320721\cdot R_{s,o}-0.50184\cdot \left[ASP\right]$$

**Figure 6:**
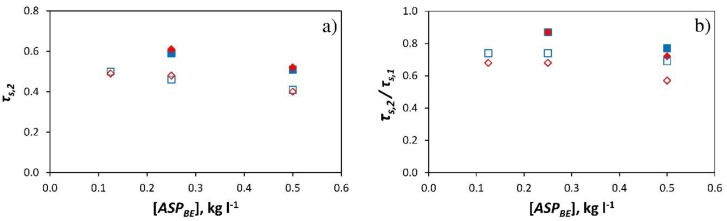
Mean values of solar transmissivity *τ_s,2_* in sector 2 with *ASP_BE_* (**a**) and of the ratio *τ_s,2_*/*τ_s,1_* (**b**) for the September-October experiments. [*ASP_BE_*], concentration in kg L^−1^. Sunny days: □, 10:00–19:00; ◊, 12:00–16:00. Cloudy and rainy days: ■, 10:00–19:00; ♦, 12:00–16:00. Subscript: *1*, sector 1 (without *ASP*); *2*, sector 2 (with *ASP*).

This fit improves on the value of R^2^ = 0.49 obtained when only the concentration of the product is considered (Figure 6a). The same fit, for the period between 12:00 and 16:00, would be (R^2^ = 0.58; *p*-value = 0.0037):

(6) $$\tau_{s,2}/\tau_{s,1}\;=0.939534-0.000199936\cdot R_{s,o}-0.545568\cdot \left[ASP\right]$$

The effect that *ASP* has in reducing the transmissivity of the greenhouse cover (*τ_s,2_*/*τ_s,1_*), decreases on days with low levels of outside radiation (days that are overcast and with a higher level of diffuse radiation) and increases with the dose of product applied. The values of R^2^ obtained in the different fits are low due to other factors on which this value depends but which are not included in the analysis, such as the variability in the concentration of product applied to the covering as a result of the method of application. However, the *p*-values below 0.05 indicate a statistically significant relationship between the variables included in the statistical analysis.

As *ASP_BE_* contains no adhesive additives, on rainy days the greenhouse cover gets “washed”. For the concentrations of 0.25 and 0.50 kg L^−1^ of the 6-day autumn experiments, the first three days were relatively clear, whereas the last three were cloudy with occasional precipitation and much lower levels of outside radiation (Table 1). As the days passed, the effect of the high atmospheric humidity, the morning dew and the showers led to a sharp fall in the effect of the product, with a concomitant increase in the transmissivity of the greenhouse cover. Comparison of the first three sunny days with the last three cloudy ones (Figure 6a) shows increases in transmissivity to solar radiation between 12:00 and 16:00 of 27% ([*ASP_BE_*] = 0.25 kg L^−1^) and 30% ([*ASP_BE_*] = 0.50 kg L^−1^), while the increases in transmissivity to PAR for the same concentrations of product were 24% and 23%, respectively. However, these increases can be attributed in part to the increase in diffuse radiation. Figure 6b illustrates that during the three cloudy days with showers the greenhouse cover is not completely washed, since the values of the ratio *τ_s,2_*/*τ_s,1_* do not reach 1.

**Figure 7:**
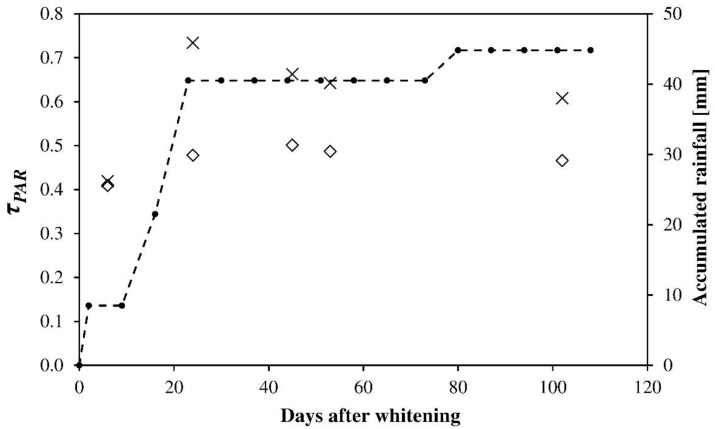
Transmissivity (to PAR radiation) of the greenhouse cover in sector 1 with *ASP_BE_* (×) and in sector 2 with *ASP_F_* (◊). Initial concentration of the product applied 25/100 (0.25 kg L^−1^). Accumulated rainfall according to data from the Almería airport weather station (-●-).

Figure 7 presents the values of transmissivity (to PAR radiation) of the greenhouse cover in sector 1 with *ASP_BE_* and in sector 2 with *ASP_F_* in spring 2015 for an initial concentration of 0.25 kg L^−1^ (25/100), together with the values of precipitation recorded at the Almería airport weather station (Almería, Spain). Transmissivity for *ASP_F_* was 0.41 six days after application, increasing to 0.48 after 24 days, several of which were rainy. In the following months, the transmissivity remained at around the same value. For *ASP_BE_*, on the other hand, transmissivity was 0.42 after six days, increasing to 0.73 after 24 days in the same meteorological conditions. This valued decreased slightly, possibly due to the accumulation of dirt on the greenhouse cover and the varied climatic conditions. The heavy rainfall at the start of the experiment washed off the *ASP_BE_* almost completely. At the conclusion of the experiment the transmissivity value for sector 1 was 0.61, similar to those in sector 1 without *ASP* recorded during the experiments in 2014 (Table 2 and Table 3).

### 3.4. Greenhouse Temperature is Influenced by the Cover with Agricultural Solar Protector

The use of whitening produced a statistically significant reduction of the temperature inside the greenhouse (Table 6) when outside mean temperature was greater than 28.5 ºC (with the exception of the *ASP_SP_* at 0.50 kg L^−1^). Whitening is traditionally used in Almeria at the end of summer and at the end of the winter, when new crops are transplanted in the greenhouse. When outside temperature begin to decrease, growers remove the whitening from cover washing it with water. When outside temperature was lower than 28.5 ºC, the whitening did not produce a significant effect in inside temperature (Table 6) whereas transmissivity to PAR radiation of the whitened cover was reduced (Table 2, Table 3, and Table 4).

**Figure 8:**
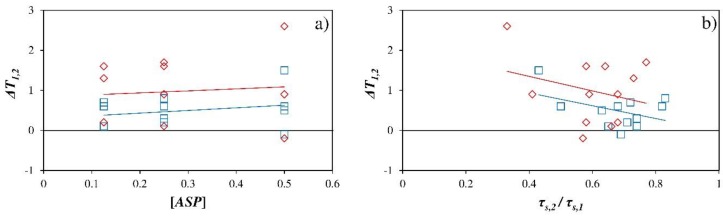
Mean values of temperature difference *ΔT_1,2_* [°C] between sector 1 (without *ASP*) and sector 2 (with *ASP*) as a function of the concentration of product applied [*ASP*] in [Kg l^−1^] (**a**) and the ratio *τ_s,2_*/*τ_s,1_* (**b**). *τ_s_*, transmissivity to solar radiation. Subscript: *1*, sector 1 (without *ASP*); *2*, sector 2 (with *ASP*). □, 10:00–19:00; ◊, 12:00–16:00.

Figure 8a illustrates that as the concentration of product applied increases there is a slight increase in temperature difference between the greenhouse sectors, although the trend is not clear due to the intrinsic variability as a result of the application method. However, it is clear that as the ratio *τ_s,2_*/*τ_s,1_* decreases, the temperature difference between sectors increases (Figure 8b).

A global analysis has been carried out considering all the products as one. It has been determined that there is a statistically significant relationship (*p*-value<0.05) between the temperature difference between the two sectors of the greenhouse on the one hand and outside solar radiation and the ratio *τ_s,2_*/*τ_s,1_* on the other; the temperature difference increases with the former and decreases with the latter. For the period 12:00–16:00 the following equation is obtained (R^2^ = 0.09; *p*-value<0.0001):

(7) $$\Delta T_{1,2}=1.01257\;+0.000994882\cdot R_{s,o}-1.40128\cdot \left[\tau_{s,2}/\tau_{s,1}\right],$$

The values of temperature difference between sector 1 (without *ASP*) and sector 2 (with *ASP*) are well below the 4.4 °C reported by Baille et al. [11], whose experiments were in a greenhouse with a crop and the transpiration rate was higher in the sector with *ASP*. However, the maximum temperature differences recorded between the two sectors at the hottest time of day, for the concentration of product recommended by the manufacturer in this province (25/100) was 4.2 °C for *ASP_BE_*, 3.9 °C for *ASP_F_*, 5.0 °C for *ASP_SF_* and 2.0 °C for *ASP_SP_*. Although no great differences were observed from 12:00 to 16:00 in the mean temperature values between sectors (Table 6), with *ASP* the maximum temperature decreases considerably inside the greenhouse without crop. This finding may prove of interest, as the conditions are similar to those of a recently transplanted crop, when plants are more sensitive to temperature extremes.

Application of *ASP* does affect the heterogeneity of temperature inside the greenhouse. The difference between the mean temperatures recorded by the “warmest” and “coldest” sensors (*ΔT_max,1_* and *ΔT_max,2_*) has been estimated for three days from 12:00 to 16:00, and it was always higher in sector 1 without *ASP* than in sector 2 with *ASP* for all four products tested (Table 6). The ratio $\sigma_{\Delta T_{i,o}}$/$\Delta T_{i,o}$ proposed by Kittas et al. [21] has also been estimated; the greater the value of this ratio, the greater the temperature heterogeneity inside the greenhouse. Table 6 shows that this ratio decreases in the sector where *ASP* is applied in 10 of the 12 experiments.

## 4. Conclusions

As final conclusions, four agricultural solar protectors (ASPs) have been tested: “Blanco de España” (*ASP_BE_*), the product traditionally used in the province of Almería, and three other commercial products that incorporate adhesives. The presence of the adhesive does not appear to influence the effect of the different products on the temperature inside the greenhouse, as all four products behave in a similar fashion at the same concentrations. The present findings support the maximum dose of product recommended by other authors: 0.50 kgL^−1^ (50/100), above which the transmissivity of the greenhouse cover produces a statistically significant decrease of over 50%. The effect of *ASP* on transmissivity of the greenhouse cover depends mainly on the dose applied, but also on the climatic conditions (solar radiation, cloud cover, etc.) and the time of year (solar elevation). This makes it difficult to recommend a single dose of product to growers. Different doses should be recommended depending on the time of year and the desired reduction in transmissivity. One of the products containing adhesives (*ASP_F_*) has been shown to remain on the greenhouse cover after periods of heavy rain, while the non-adhesive product traditionally used (*ASP_BE_*) is washed away. The method of application of *ASP* should be standardised in order to establish a means of applying a given concentration of product in gm^−2^ of cover. The traditional method of application establishes a dose (in kgL^−1^), but the amount of product that finally remains on the cover is impossible to determine as it is applied manually.

## Figures and Tables

**Table 1:** Mean daily values of outside climatic conditions on test dates: *DOY*, day of year; *u_o_*, wind velocity [m s^−1^]; *θ*, wind direction [°]; *RH_o_*, relative air humidity [%]; *T_o_*, air temperature [°C]; *R_s,o_*, outside solar radiation [W m^−2^].

*ASP*	[kg L^−1^]	Date	*DOY*	*u_o_*	*θ* ^a^	*RH_o_*	*T_o_*	*R_s,o_*
*BE* *“Blanco España”*	0.08	19–21/07/2014	198–200	2.9 ± 0.7	210.7 ± 65.3	63.4 ± 11.8	24.8 ± 1.8	338.6 ± 8.1
	0.125	23, 25–26/07/2014	202–205	1.6 ± 0.5	198.2 ± 6.9	62.2 ± 17.9	26.5 ± 0.7	327.8 ± 17.7
	0.25	29–31/07/2014	208–210	2.9 ± 0.5	190.2 ± 44.0	72.0 ± 9.3	26.0 ± 1.4	301.0 ± 50.6
	0.50	02–04/08/2014	212–214	1.9 ± 0.9	196.3 ± 30.8	62.4 ± 8.4	23.3 ± 0.8	326.3 ± 8.3
*F* *Flex*	0.125	10–12/08/2014	219–221	1.3 ± 0.5	197.5 ± 24.8	76.1 ± 3.2	25.2 ± 0.4	317.9 ± 16.4
	0.25	14–16/08/2014	223–225	4.3 ± 1.5	123.4 ± 33.6	67.1 ± 4.7	28.1 ± 1.0	274.0 ± 18.2
	0.50	20–22/08/2014	229–231	1.7 ± 0.1	187.3 ± 18.7	75.8 ± 3.8	25.1 ± 0.7	314.1 ± 7.5
*SF* *SuperFlex*	0.125	24–26/08/2014	233–235	2.0 ± 1.0	172 ± 61.7	70.5 ± 8.1	26.0 ± 1.4	312.3 ± 0.6
	0.25	28–30/08/2014	237–239	2.1 ± 0.9	165.2 ± 49.0	71.8 ± 8.3	27.5 ± 0.7	281.9 ± 28.2
	0.50	02–04/09/2014	241–243	1.3 ± 0.1	198.1 ± 22.3	76.6 ± 8.5	26.2 ± 0.5	292.3 ± 4.3
*EP* *Special pepper*	0.125	06–08/09/2014	245–247	1.6 ± 0.5	233.5 ± 33.7	75.9 ± 2.0	25.2 ± 0.3	266.2 ± 5.8
	0.25	10–12/09/2014	249–251	2.1 ± 1.0	217.3 ± 45.5	74.6 ± 2.7	24.0 ± 0.8	267.2 ± 6.0
	0.50	14–16/09/2014	253–255	1.6 ± 0.3	191.4 ± 19.8	73.1 ± 8.9	22.1 ± 0.3	214.1 ± 95.1
*BE* *“Blanco España”*	0.125	18–20/09/2014	257–259	1.7 ± 1.1	212.4 ± 18.5	69.5 ± 2.5	22.2 ± 0.4	185.9 ± 25.9
	0.25	24–26/09/2014	263–265	2.7 ± 1.8	144.8 ± 57.2	73.6 ± 8.2	22.0 ± 2.1	228 ± 19.1
	0.25 *	27–29/09/2014	266–268	3.0 ± 2.4	124.3 ± 49.2	76.4 ± 6.2	22.0 ± 0.5	100.3 ± 24.2
	0.50	07–09/10/2014	276–278	1.3 ± 0.3	223.0 ± 20.0	82.9 ± 2.2	20.1 ± 0.5	228.6 ± 4.2
	0.50 *	10–12/10/2014	279–281	2.7 ± 1.1	226.7 ± 76.0	76.2 ± 3.1	20.8 ± 1.0	147.9 ± 43.4

^a^ Wind direction perpendicular to the roof vents is 208° for southwesterly *Poniente* winds and 28° for the northeasterly *Levante* winds. *Replications carried out on overcast days with occasional light showers.

**Table 2:** Mean values of transmissivity of the greenhouse cover in sector 1 (without *ASP_BE_*) and sector 2 (with *ASP_BE_*) for the experiments carried out in summer. *DOY*, day of year at the beginning of the test; [*ASP_BE_*], concentration in kg L^−1^; *τ_s_*, transmissivity to solar/global radiation; *τ_PAR_*, transmissivity to PAR; *R_s_*, incoming solar radiation above the crop; *R_PAR_*, PAR radiation; *Rn*, net radiation. Subscript: *1*, inside sector 1 (without *ASP_BE_*); *2*, inside sector 2 (with *ASP_BE_*); *o*, outside.

*DOY*	[*ASP*_BE_]	*τ_s,1_*	*τ_s,2_*	*τ_PAR,1_*	*τ_PAR,2_*	*R_n,1_/R_s,1_*	*R_n,2_/R_s,2_*	*R_PAR,o_/R_s,o_*	*R_PAR,1_/R_s,1_*	*R_PAR,2_/R_s,2_*
	10:00–19:00									
198	0.08	0.57 ± 0.09 ^f^	0.46 ± 0.03 ^d^	0.55 ± 0.07 ^e^	0.43 ± 0.03 ^d^	0.57	0.53	0.46	0.45	0.43
202	0.125	0.57 ± 0.08 ^f^	0.41 ± 0.04 ^c^	0.55 ± 0.06 ^e^	0.39 ± 0.03 ^c^	0.56	0.50	0.45	0.45	0.43
208	0.25 *	0.59 ± 0.10 ^e^	0.37 ± 0.03 ^b^	0.58 ± 0.07 ^f^	0.34 ± 0.03 ^b^	0.57	0.49	0.46	0.45	0.42
211	0.50	0.59 ± 0.10 ^e^	0.33 ± 0.03 ^a^	0.58 ± 0.08 ^f^	0.30 ± 0.03 ^a^	0.56	0.48	0.46	0.45	0.42
	12:00–16:00									
198	0.08	0.62 ± 0.05 ^e^	0.47 ± 0.01 ^d^	0.62 ± 0.03 ^f^	0.45 ± 0.01 ^d^	0.63	0.58	0.46	0.45	0.43
202	0.125	0.61 ± 0.04 ^e^	0.40 ± 0.01 ^c^	0.60 ± 0.03 ^e^	0.38 ± 0.01 ^c^	0.61	0.56	0.45	0.45	0.43
208	0.25 *	0.66 ± 0.06 ^f^	0.37 ± 0.01 ^b^	0.64 ± 0.04 ^g^	0.34 ± 0.01 ^b^	0.63	0.56	0.46	0.45	0.43
211	0.50	0.66 ± 0.06 ^f^	0.32 ± 0.01 ^a^	0.65 ± 0.03 ^h^	0.30 ± 0.01 ^a^	0.61	0.56	0.45	0.44	0.42

* Data from the first day of experimentation, which was overcast, were omitted. ^a–h^ Values of transmissivity accompanied by different letters are significantly different at 95.0% confidence level (*p*-value < 0.05) for each time period (10:00–19:00 or 12:00–16:00).

**Table 3:** Mean values of transmissivity of the greenhouse cover in sector 1 (without *ASP_BE_*) and sector 2 (with *ASP_BE_*) for the autumn experiments. *DOY*, day of year; [*ASP_BE_*], concentration in kg L^−1^; *τ_s_*, transmissivity to solar radiation; *τ_PAR_*, transmissivity to PAR; *R_s_*, inside solar radiation; subscript: *1*, sector 1 (without *ASP_BE_*); *2*, sector 2 (with *ASP_BE_*).

*DOY*	[*ASP_BE_*]	*τ_s,1_*	*τ_s,2_*	*τ_PAR,1_*	*τ_PAR,2_*
	10:00–19:00				
257–259	0.125	0.68 ± 0.06 ^f^	0.50 ± 0.04 ^c^	0.62 ± 0.05 ^e^	0.42 ± 0.04 ^a^
263–265	0.25	0.62 ± 0.11 ^e^	0.46 ± 0.05 ^b^	0.63 ± 0.10 ^d^	0.44 ± 0.07 ^b^
276–278	0.50	0.59 ± 0.15 ^d^	0.41 ± 0.05 ^a^	0.65 ± 0.13 ^c^	0.42 ± 0.10 ^a^
	12:00–16:00				
257–259	0.125	0.72 ± 0.05 ^d^	0.49 ± 0.04 ^c^	0.64 ± 0.03 ^d^	0.41 ± 0.04 ^b^
263–265	0.25	0.71 ± 0.07 ^d^	0.48 ± 0.04 ^b^	0.70 ± 0.07 ^e^	0.45 ± 0.05 ^c^
276–278	0.50	0.70 ± 0.11 ^d^	0.40 ± 0.04 ^a^	0.74 ± 0.11 ^f^	0.39 ± 0.04 ^a^

^a–f^ Values of transmissivity accompanied by different letters are significantly different at 95.0% confidence level (*p*-value < 0.05) for each time period (10:00–19:00 or 12:00–16:00).

**Table 4:** Mean values of transmissivity of the greenhouse cover in sector 1 (without *ASP*) and sector 2 (with *ASP*) for the products with adhesive. *DOY*, day of year; [*ASP*], concentration of product in kg L^−1^; *τ_s_*, transmissivity to solar radiation; *τ_PAR_*, transmissivity to PAR; subscript: *1*, sector 1 (without *ASP*); *2*, sector 2 (with *ASP*). Products: *ASP_F_*, flex; *ASP_SF_*, superflex; *ASP_SP_*, special pepper.

***DOY***	**[*ASP_F_*]**	***τ_s,1_***	***τ_s,2_***	***τ_PAR,1_***	***τ_PAR,2_***
	10:00–19:00				
219–221	0.125	0.57 ± 0.12 ^d^	0.47 ± 0.03 ^b^	0.56 ± 0.08 ^d^	0.44 ± 0.03 ^b^
223–225	0.25*	0.63 ± 0.12 ^e^	0.52 ± 0.07 ^c^	0.61 ± 0.09 ^e^	0.48 ± 0.06 ^c^
229–231	0.50	0.61 ± 0.11 ^e^	0.26 ± 0.03 ^a^	0.59 ± 0.08 ^e^	0.24 ± 0.03 ^a^
	12:00–16:00				
219–221	0.125	0.66 ± 0.09 ^d^	0.48 ± 0.03 ^b^	0.62 ± 0.06 ^d^	0.44 ± 0.02 ^b^
223–225	0.25*	0.69 ± 0.12 ^f^	0.53 ± 0.09 ^c^	0.66 ± 0.10 ^f^	0.49 ± 0.07 ^c^
229–231	0.50	0.69 ± 0.07 ^e^	0.23 ± 0.01 ^a^	0.65 ± 0.05 ^e^	0.21 ± 0.01 ^a^
***DOY***	**[*ASP_SF_*]**	***τ_s,1_***	***τ_s,2_***	***τ_PAR,1_***	***τ_PAR,2_***
	10:00–19:00				
233–235	0.125	0.61 ± 0.11 ^d^	0.44 ± 0.05 ^c^	0.59 ± 0.08 ^d^	0.42 ± 0.04 ^c^
237–239	0.25*	0.62 ± 0.10 ^d^	0.42 ± 0.05 ^b^	0.60 ± 0.08 ^d^	0.39 ± 0.07 ^b^
241–243	0.50	0.62 ± 0.09 ^d^	0.31 ± 0.03 ^a^	0.59 ± 0.08 ^d^	0.28 ± 0.03 ^a^
	12:00–16:00				
233–235	0.125	0.70 ± 0.06 ^e^	0.45 ± 0.06 ^c^	0.65 ± 0.04 ^d^	0.42 ± 0.04 ^c^
237–239	0.25*	0.69 ± 0.07 ^d^	0.40 ± 0.05 ^b^	0.65 ± 0.06 ^d^	0.36 ± 0.05 ^b^
241–243	0.50	0.69 ± 0.07 ^d^	0.28 ± 0.01 ^a^	0.65 ± 0.05 ^d^	0.25 ± 0.01 ^a^
***DOY***	**[*ASP_SP_*]**	***τ_s,1_***	***τ_s,2_***	***τ_PAR,1_***	***τ_PAR,2_***
	10:00–19:00				
245–247	0.125	0.66 ± 0.11 ^e^	0.43 ± 0.03 ^b^	0.59 ± 0.08 ^c^	0.36 ± 0.03 ^b^
249–251	0.25	0.65 ± 0.12 ^d,e^	0.47 ± 0.05 ^c^	0.60 ± 0.09 ^d^	0.40 ± 0.05 ^c^
253–255	0.50*	0.65 ± 0.09 ^e^	0.40 ± 0.04 ^a^	0.61 ± 0.07 ^e^	0.34 ± 0.04 ^a^
	12:00–16:00				
245–247	0.125	0.74 ± 0.10 ^e^	0.43 ± 0.03 ^b^	0.65 ± 0.06 ^d^	0.36 ± 0.03 ^c^
249–251	0.25	0.73 ± 0.08 ^e^	0.48 ± 0.05 ^c^	0.66 ± 0.05 ^e^	0.41 ± 0.04 ^b^
253–255	0.50*	0.70 ± 0.06 ^d^	0.41 ± 0.04 ^a^	0.66 ± 0.05 ^d^	0.35 ± 0.04 ^a^

* Partially overcast days. ^a–f^ Values of transmissivity accompanied by different letters are significantly different at 95.0% confidence level (*p*-value < 0.05) for each time period (10:00–19:00 or 12:00–16:00).

**Table 5:** Mean values of ratio ***τ_s,2_/τ_s,1_*** of transmissivity to solar radiation of the greenhouse cover in sector 2 ***τ_s,2_*** (with *ASP*) and sector 1 ***τ_s,1_*** (without *ASP*) for each concentration *[ASP]* in kg L^−1^.

[*ASP*]	*ASP_BE_*	*ASP_F_*	*ASP_SF_*	*ASP_SP_*	*ASP_BE_*
	10:00–19:00				
0.125	0.72 ^h^	0.82 ^j^	0.72 ^h^	0.65 ^f^	0.74 ^i^
0.25	0.63 ^e,f^	0.83 ^j^	0.68 ^g^	0.72 ^h^	0.74 ^i^
0.50	0.56 ^c^	0.43 ^a^	0.50 ^b^	0.62 ^d^	0.69 ^g,h^
	12:00–16:00				
0.125	0.66 ^h^	0.73 ^j^	0.64 ^g^	0.58 ^e^	0.68 ^i^
0.25	0.56 ^d,e^	0.77 ^k^	0.58 ^e^	0.66 ^h^	0.68 ^i^
0.50	0.48 ^c^	0.33 ^a^	0.41 ^b^	0.59 ^e,f^	0.57 ^e^

^a–k^ Values accompanied by different letters are significantly different at 95.0% confidence level (*p*-value < 0.05) for each time period (10:00–19:00 or 12:00–16:00).

**Table 6:** Mean outside air temperature *T_0_* [°C]; mean temperatures inside sector 1 (without *ASP*) *T_1_* and sector 2 (with *ASP*) *T_2_* [°C]; ]; maximum difference between the mean temperatures inside sectors 1 and 2 *ΔT_1,2 max_* [°C]; temperature difference between sector 2 (with *ASP*) and outside *ΔT_2,o_* [°C]; maximum difference between the mean temperatures recorded by the different sensors in sectors 1 and 2, *ΔT_max,1_* and *ΔT_max,2_* [°C]; ratio for the heterogeneity of temperature distribution inside the greenhouse $\sigma_{\Delta T_{i,o}}$/$\Delta T_{i,o}$,. Values for the time period 12:00–16:00.

[*ASP*]	*T_0_*	*T_1_*	*T_2_*	*ΔT_1,2 max_*	*ΔT_2,o_*	*ΔT_max,1_*	*ΔT_max,2_*	$\sigma_{\Delta T_{1,o}}$ $/\Delta T_{1,o}\;$	$\sigma_{\Delta T_{2,o}}$ $/\Delta T_{2,o}$
	[*ASP_F_*]								
0.125	28.6 ± 0.9	35.5 ± 1.8 ^b^	34.2 ± 1.7 ^a^	3.0	5.6	3.4	2.8	0.177	0.183
0.25	32.5 ± 1.5	41.6 ± 2.7 ^b^	39.9 ± 2.3 ^a^	3.9	7.4	1.8	2.3	0.085	0.122
0.50	29.4 ± 2.7	37.3 ± 3.7 ^b^	34.7 ± 2.9 ^a^	5.5	5.3	3.8	2.5	0.182	0.179
	*[ASP_SF_*]								
0.125	30.5 ± 2.6	38.5 ± 3.4 ^b^	36.9 ± 2.8 ^a^	4.2	6.4	3.4	2.3	0.165	0.139
0.25	31.9 ± 2.8	39.5 ± 3.8 ^b^	37.9 ± 3.1 ^a^	5.0	6.0	3.0	2.8	0.154	0.184
0.50	29.5 ± 0.5	36.4 ± 0.9 ^b^	35.5 ± 0.9 ^a^	3.6	6.0	4.1	2.1	0.208	0.136
	[*ASP_SP_*]								
0.125	28.2 ± 0.6	34.1 ± 0.8 ^a^	33.9 ± 0.8 ^a^	2.0	5.7	3.4	2.2	0.203	0.146
0.25	27.6 ± 0.8	33.8 ± 1.3 ^a^	33.9 ± 1.3 ^a^	2.6	6.3	3.5	2.3	0.195	0.139
0.50	26.0 ± 1.0	31.8 ± 1.8 ^b^	30.9 ± 2.0 ^a^	3.0	4.9	2.9	1.7	0.166	0.135
	[*ASP_BE_*] autumn								
0.125	25.4 ± 1.4	31.2 ± 2.6 ^a^	31.4 ± 3.0 ^a^	2.0	6.0	3.2	2.2	0.193	0.135
0.25	26.1 ± 3.7	31.9 ± 4.6 ^a^	31.0 ± 3.6 ^a^	4.2	4.9	2.7	1.8	0.188	0.163
0.50	23.9 ± 0.8	29.9 ± 1.2 ^a^	29.7 ± 1.1 ^a^	2.0	5.8	3.7	2.3	0.219	0.140

^a,b^ Values of temperature accompanied by different letters are significantly different at 95.0% confidence level (*p*-value < 0.05) for each concentration.

## References

[1] Valera D.L., Belmonte L.J., Molina-Aiz F.D., López A. (2016). Greenhouse Agriculture in Almería. A Comprehensive Techno-Economic Analysis.

[2] Reyes-Rosas A., Molina-Aiz F.D., Valera D.L., López A., Khamkure S. (2017). Development of a single energy balance model for prediction of temperatures inside a naturally ventilated greenhouse with polypropylene soil mulch. Comput. Electron. Agric..

[3] Rodriguez F., Berenguel M., Guzman J.L., Ramírez-Arias A. (2015). The greenhouse dynamic system. Modeling and Control of Greenhouse Crop Growth.

[4] Chauhan P.M., Kim W.S., Lieth J.H. Combined effect of whitening and ventilation methods on microclimate and transpiration in rose greenhouse. Proceedings of the International Conference on Thermal Energy Storage Technologies, Devi Ahilya University.

[5] López-Marín J., González A., Gálvez A. (2011). Effect of shade on quality of greenhouse peppers. Acta Hortic..

[6] Abdel-Ghany A.M., Picuno P., Al-Helal I., Alsadon A., Ibrahim A., Shady M. (2015). Radiometric characterization, solar and thermal radiation in a greenhouse as affected by shading configuration in an arid climate. Energies.

[7] Katsoulas N., Kittas C. (2008). Impact of greenhouse microclimate on plant growth and development with special reference to the *Solanaceae*. Eur. J. Plant Sci. Biotechnol..

[8] Gázquez J.C., López J.C., Pérez-Parra J.J., Baeza E.J., Lorenzo P., Caparros I. (2012). Effects of three cooling systems on the microclimate of a greenhouse with a pepper crop in the Mediterranean area. Acta Hortic..

[9] Kittas C., Baille A., Giaglaras P. (1999). Influence of cover material and shading on the spectral distribution of light in greenhouses. J. Agric. Eng. Res..

[10] Fernández E.J., Fernández J., Camacho F., Vázquez J.J., Kenig A. (2000). Radiative field uniformity under shading screens under greenhouse vs. whitewash in Spain. Acta Hortic..

[11] Baille A., Kittas C., Katsoulas N. (2001). Influence of whitening on greenhouse microclimate and crop energy partitioning. Agric. For. Meteorol..

[12] Abreu P.E., Meneses J.F. (2000). Influence of soil covering, plastic ageing and roof whitening on climate and tomato crop response in an unheated plastic Mediterranean greenhouse. Acta Hortic..

[13] Goudriaan G., van Laar H.H. (1994). Modelling Potential Crop Growth Processes.

[14] Luo W., Stanghellini C., Dai J., Wang X., de Zwart H.F., Bu C. (2005). Simulation of greenhouse management in the subtropics, part II: Scenario study for the summer season. Biosyst. Eng..

[15] Mashonjowa E., Ronsse F., Mhizha T., Milford J.R., Lemeur R., Pieters J.G. (2010). The effects of whitening and dust accumulation on the microclimate and canopy behaviour of rose plants (*Rosa hybrida*) in a greenhouse in Zimbabwe. Sol. Energy.

[16] López A., Molina-Aiz F.D., Valera D.L., Peña A. (2016). Wind tunnel analysis of the airflow through insect-proof screens and comparison of their effect when installed in a mediterranean greenhouse. Sensors.

[17] Statgraphics (2017). Statgraphics^®^ Centurion 18. Manual de Usuario.

[18] Hanan J.J. (1998). Greenhouses: Advanced Technology for Protected Horticulture.

[19] McCree K.J. (1972). Test of current definitions of photosynthetically active radiation against leaf photosynthesis data. Agric. Meteorol..

[20] Soriano T., Montero J.I., Sánchez-Guerrero M.C., Medrano E., Antón A., Hernández J., Morales M.I., Castilla N. (2004). A study of direct solar radiation transmission in asymmetrical multi-span greenhouses using scale models and simulation models. Biosyst. Eng..

[21] Kittas C., Katsoulas N., Bartzanas T., Mermier M., Boulard T. (2008). The impact of insect screens and ventilation openings on the greenhouse microclimate. Trans. Asabe.

